# Profiling the Urinary Microbiota in Male Patients With Bladder Cancer in China

**DOI:** 10.3389/fcimb.2018.00167

**Published:** 2018-05-31

**Authors:** Peng Wu, Guihao Zhang, Jie Zhao, Jiawei Chen, Yang Chen, Weina Huang, Jialei Zhong, Jiarong Zeng

**Affiliations:** ^1^Department of Urology, Nanfang Hospital, Southern Medical University, Guangzhou, China; ^2^School of Pharmaceutical Sciences, Southern Medical University, Guangzhou, China

**Keywords:** urinary bladder neoplasms, urinary tract, microbiota, extracellular matrix, inflammation

## Abstract

Mounting evidence indicates that microbiome plays an important role in the development and progression of cancer. The dogma that urine in healthy individuals must be sterile has been overturned. Dysbiosis of the urinary microbiome has been revealed responsible for various urological disorders, including prostate cancer. The link between chronic inflammation, microbiome and solid tumors has been established for various neoplastic diseases. However, a detailed and comprehensive analysis of urinary microenvironment of bladder cancer has not been yet reported. We performed this study to characterize the potential urinary microbial community possibly associated with bladder cancer. Mid-stream urine was collected from 31 male patients with bladder cancer and 18 non-neoplastic controls. DNA was extracted from urine pellet samples and processed for high throughput 16S rRNA amplicon sequencing of the V4 region using Illumina MiSeq. Sequencing reads were filtered using QIIME and clustered using UPARSE. We observed increased bacterial richness (Observed Species, Chao 1 and Ace indexes; cancer vs. control; 120.0 vs. 56.0; 134.5 vs. 68.3; and 139.6 vs. 72.9, respectively), enrichment of some bacterial genera (e.g., *Acinetobacter, Anaerococcus, and Sphingobacterium*) and decrease of some bacterial genera (e.g., *Serratia, Proteus*, and *Roseomonas*) in cancer group when compared to non-cancer group. Significant difference in beta diversity was found between cancer and non-cancer group, among different risk level, but not among different tumor grade. Enrichment of *Herbaspirillum, Porphyrobacter*, and *Bacteroides* was observed in cancer patients with high risk of recurrence and progression, which means these genera maybe potential biomarkers for risk stratification. The PICRUSt showed that various functional pathways were enriched in cancer group, including *Staphylococcus aureus* infection, glycerolipid metabolism and retinol metabolism. To our knowledge, we performed the most comprehensive study to date to characterize the urinary microbiome associated with bladder cancer. A better understanding of the role of microbiome in the development and progression of bladder cancer could pave a new way for exploring new therapeutic options and biomarkers.

## Introduction

Bladder cancer is diagnosed in more than 430,000 patients worldwide every year, making it the ninth most common malignancy (Kamat et al., 2016). In addition, most of bladder cancer patients are male, five to six times more common than female in China (Li et al., 2015). In the past decades, bladder cancer has aroused scientists' attention for its high morbidity and mortality rates. Unfortunately, the etiology and pathophysiology of bladder cancer remain unknown. It may be caused by genetic mutations and external risk factors, including tobacco smoking, carcinogen exposure, the chlorination of drinking water and possibly cyclophosphamide (Babjuk et al., 2017).

Notably, all above factors are known to affect the composition of microbiota, the ensemble of symbiotic bacteria, fungi, parasites, and viruses that inhabit the epithelial barrier surfaces of our body (Costello et al., 2012). The microbiome affects human physiological functions, such as metabolism, immunity and haematopoiesis (Dzutsev et al., 2015). In addition, the microbiome also plays a role in the development of malignancies both at epithelial barriers and in tissues (Roy and Trinchieri, 2017). Studies suggest that microbial dysbiosis at various body sites may promote disease progression, such as periodontitis, inflammatory bowel disease, colorectal cancer and breast cancer (Darveau, 2010; Irrazábal et al., 2014; Chu et al., 2016; Urbaniak et al., 2016). Recently, emerging evidence overturns the dogma that urine in healthy individuals must be sterile (Whiteside et al., 2015). Furthermore, dysbiosis of the urinary microbiome has been revealed responsible for various urological disorders, such as urgency urinary incontinence, interstitial cystitis, overactive bladder and prostate cancer (Siddiqui et al., 2012; Pearce et al., 2014; Wu et al., 2017; Shrestha et al., 2018).

Accordingly, it is conceivable that alteration of urinary microbiome may be associated with bladder cancer. Although the link between specific pathogens and cancer is well established, such as *Helicobacter pylori* and gastric cancer (El-Omar et al., 1997), there is currently no hard evidence linking microbiome and bladder cancer. Intravesical instillation of *Mycobacterium bovis* bacillus Calmette–Guérin and oral administration of *Lactobacillus* after removal of the bladder tumor could reduce the probability of recurrence (Aso et al., 1995; Zitvogel et al., 2017). Dysbiosis caused by repeated antibiotic use can increase the incidence of cancers, including bladder cancer (Boursi et al., 2015). In addition, a case-control study showed that regular probiotic intake reduced the risk of bladder cancer in the healthy population (Ohashi et al., 2002). Taken together, these results strongly support the hypothesis that microbiome might be involved in bladder carcinogenesis, progression and relapse.

However, despite mounting researches on the human microbiome have yielded multiple insights into health and disease including cancers (Thomas et al., 2016), a detailed and comprehensive analysis of microbiota in urine of bladder cancer has not been yet reported. A recent study suggested that microbiome may be a factor in bladder cancer pathology and further studies on the urinary microbiota of bladder cancer would direct urologists to new therapeutic and prognostic options (Bucevic Popovic et al., 2017). Our primary purpose was to characterize urinary microbiota associated with bladder cancer in China and to explore the role of microbiome in bladder carcinogenesis.

## Materials and methods

### Subject recruitment and specimen collection

Urine specimens were collected from male patients with bladder cancer and non-neoplastic patients admitted to Nanfang Hospital in China between March 2017 and September 2017. All cancer cases were histologically confirmed as urothelial carcinoma and male controls were cases for a wide spectrum of non-neoplastic conditions, such as renal cyst. Subjects with prior known sexually transmitted infection or a recent history of urinary tract infections or antibiotic usage for any indication (within 1 month) were excluded. All subjects were required to finish a structured questionnaire to collect information on socio-demographic characteristics. Data collection followed the principles outlined in the Declaration of Helsinki. All participants had signed a written informed consent to contribute their own anonymous information to this study. Our study was approved by the Medicine Institutional Review Board of Southern Medical University.

Mid-stream urine specimens were collected by the clean catch method under the guidance of urotherapy nurses, then centrifuged at 16,000 g for 10 min immediately and stored at −80°C until further processing.

### DNA isolation and 16s rRNA gene sequencing

To avoid contamination, DNA isolation was performed using the cultured cells protocol supplied with the DNeasy Blood and Tissue Kit (Qiagen, Germany) in a laminar flow hood. The concentration of extracted DNA was determined through a Nanodrop ND-1000 spectrophotometer (Thermo Electron Corporation, USA). The genomic DNA isolated from the clinical samples was amplified using primer sets specific for V4 regions (515F: GTGCCAGCMGCCGCGGTAA; and 806R:GGACTACHVGGGTWTCTAAT). In order to evaluate contribution of extraneous DNA from reagents, extraction negative controls (no urine) and PCR negative controls (no template) were included. The resultant PCR products were purified by Qiaquick PCR purification kit (Qiagen, Valencia, CA). Finally, purified samples were normalized to equal DNA concentration and sequenced using the Illumina Miseq sequencer (Illumina, Inc., USA). The 16S rRNA gene sequences have been submitted to the Short Read Archive (SRA) under accession number SUB3915640.

### Bioinformatics analysis

Raw data were filtered to eliminate reads with adapter pollution and low quality to obtain clean reads by using QIIME (Caporaso et al., 2010). Filtered sequences were clustered by 97% identity into operational taxonomic units (OTUs) using UPARSE (Edgar, 2013), and subsequently, a single representative sequence from each clustered OTU was used to align to the SILVA database (Quast et al., 2013) and the Greengenes database (DeSantis et al., 2006) by Ribosomal Database Project Classifier (Wang et al., 2007).

QIIME was used to evaluate alpha diversity, which is composed of the Observed Species, Chao1, Shannon, Simpson and Ace indexes. Among them, the Observed Species, Chao1 and Ace indexes are indicators of species richness, while Shannon and Simpson indexes are indicators of species diversity. The difference of alpha diversity between groups was evaluated by Wilcoxon Rank-Sum Test (group number = 2) and Kruskal–Wallis test (*n* > 2) using SPSS (version 22).

To compare microbial composition between groups, beta diversity was evaluated by calculating the Bray Curtis, weighted UniFrac and unweighted UniFrac distances. Principal coordinate analysis (PCoA) was applied to generate three-dimensional plots in QIIME based on these distance matrices. The PERMANOVA was performed to test for statistical significance between groups using 999 permutations in QIIME.

Differential abundance analysis between groups was performed using Metastats and *P*-values were adjusted for multiple hypothesis testing using the False Discovery Rate based on the Benjamini-Hochberg (White et al., 2009). To identify significantly different bacteria between groups, taxa summaries were reformatted and input into Linear discriminant analysis effect size (LEfSe) via the Huttenhower Lab Galaxy Server (Segata et al., 2011). The Kruskal-Wallis rank sum test and Wilcoxon test were used to identify biomarkers, and linear discriminant analysis (LDA) was used to score them. Only taxa with logarithmic LDA score greater than 2 at a *P* < 0.05 were considered significantly enriched. To predict the functional pathways from microbiota composition data, Phylogenetic Investigation of Communities by Reconstruction of Unobserved States (PICRUSt) was performed for reconstruction of metagenome (Langille et al., 2013). Predicted functional genes were categorized into Kyoto Encyclopedia of Genes and Genomes (KEGG) orthology and compared across patient groups using STAMP (version 2.1.3, Parks et al., 2014).

### Statistical analysis

Data are presented as median (first quartile to the third quartile) for continuous variables or number of cases (%) for counts data. The statistical significance of differences between groups were evaluated using Mann-Whitney U-test for continuous variables and Pearson's chi-square test or Fisher's Exact Test for count data through SPSS software (Version 22.0). All tests were two sided and *P* < 0.05 were considered statistically significant. PASS programme (PASS 11, NCSS, Kaysville UT, USA) provides estimates of power by simulation. Estimates were obtained 2000 simulations. We estimated that with 31 patients in cancer group and 18 subjects in control group, we would have 90% power to detect differences at the 0.05 significance level (alpha) using a two-sided Mann-Whitney Test.

## Results

### Subjects and samples characteristics

A total of 60 samples were analyzed, including 35 from male cancer patients and 25 from male non-neoplastic controls, while 4 samples in cancer group and 7 samples in control group were excluded for samples with too little sequencing reads (Supplementary Table 1). Detailed information on clinical characteristics and pathological parameters can be found in Table 1 and Supplementary Table 1. No significant difference was observed in the demographic characteristics between cancer and non-cancer group, except cigarette smoking (Table 1).

**Table 1:** Comparisons of demographic characteristics and parameter of alpha diversity between cancer patients and non-cancer controls.

	**Cancer (*n* = 31)**	**Non-cancer (*n* = 18)**	***P*-value**
**DEMOGRAPHIC**			
Age (y)	64.0 (49.0, 69.0)	55.5 (45.8, 64.8)	Ns
BMI	22.9 (20.8, 24.2)	22.1 (20.1, 23.4)	Ns
Smoking habit	24 (77.4)	7(38.9)	0.007
Smoking index	600 (50, 800)	150 (0, 300)	0.002
Drinking habit	8 (25.8)	8 (44.4)	Ns
Hypertension	10 (32.3)	4 (22.2)	Ns
Diabetes	6 (18.2)	1(5.6)	Ns
Hyperlipidemia	2 (6.5)	2 (11.1)	Ns
CHD	3 (9.7)	1 (5.6)	Ns
FHC	1 (3.0)	0 (0)	Ns
**ALPHA DIVERSITY**			
Number of reads	105440.0 (83300.0, 107694.0)	98289.0 (60820.0, 107601.0)	Ns
Observed species	120.0 (77.0, 147.0)	56.0 (49.0, 107.3)	0.008
Chao1	134.5 (82.5, 172.4)	68.3 (54.4, 116.8)	0.008
Ace	139.6 (41650.0, 53847.0)	72.9 (54.9, 111.9)	0.003
Shannon	2.4 (1.6, 2.9)	2.3 (1.8, 2.8)	Ns
Simpson	0.2 (0.1, 0.4)	0.2 (0.1, 0.3)	Ns

*Data were presented as median (first quartile to the third quartile) for continuous variables or n (%) for counts. BMI, body mass index; CHD, coronary atherosclerotic heart disease; FHC, family history of cancer; Ns, not significant (based on P < 0.05)*.

Cancer group was composed of 26 patients with non-muscle-invasive bladder cancer (NMIBC) and 5 patients with muscle-invasive bladder cancer (MIBC). The 2004 WHO grading system categorized cancer group as papillary urothelial neoplasm of low malignant potential (PUNLMP, *n* = 5) or low-grade papillary urothelial carcinoma group (LG, *n* = 11) or high-grade papillary urothelial carcinoma group (HG, *n* = 15) (Supplementary Table 2). Based on European Organization for Research and Treatment of Cancer (EORTC) scoring system (Lughezzani et al., 2011) (Supplementary Table 3), NMIBC group were stratified into lower risk of recurrence group (LER, recurrence score of EORTC ≤ 4, *n* = 16) and higher risk of recurrence group (HER, recurrence score of EORTC ≥ 5, *n* = 10), lower risk of progression group (LEP, progression score of EORTC ≤ 6, *n* = 15) and higher risk of progression group (HEP, progression score of EORTC ≥ 7, *n* = 11), respectively (Supplementary Table 2).

### Sequencing data, alpha, and beta diversity

**Figure 1:**
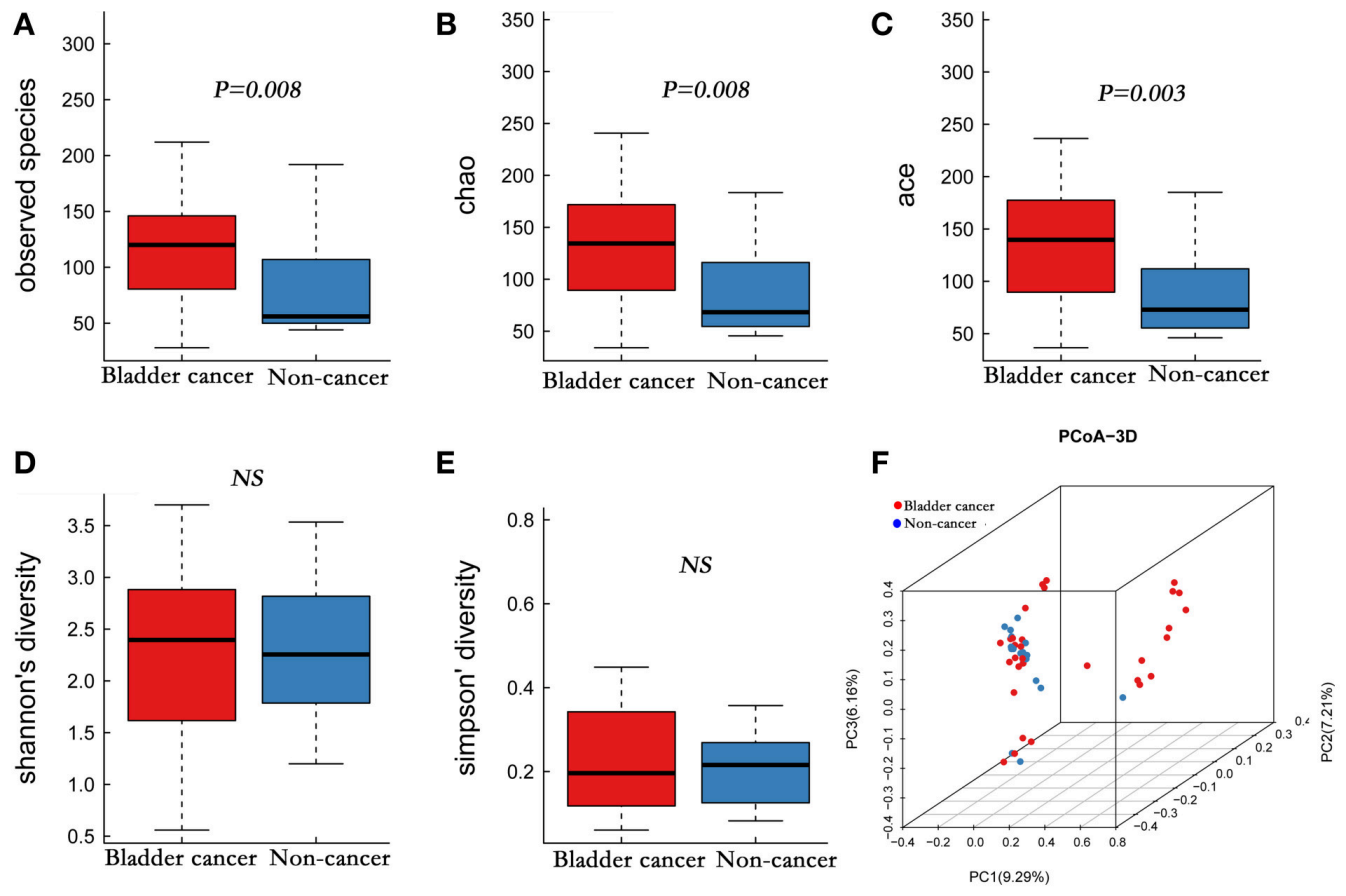
Alpha and principal coordinate analysis (PCoA) for bladder-cancer samples and non-cancer samples. **(A–E)** Box-plot showing alpha diversity in samples using different metrics (**A**, observed species index; **B**, Chao1 index; **C**, Ace index; **D**, Shannon index; **E**, Simpson index). **(F)** PCoA plots of unweighed UniFrac distances in which samples were colored by clinical outcome. The PERMANOVA performed on the unweighted UniFrac distances showed that the observed differences were statistically significant (999 permutations; *F* = 2.53; *P* < 0.001).

A total of 4,427,184 clean reads were obtained from the 49 samples. The median number of reads in cancer patients was 105,440, and in the non-cancer patients was 98,289 (Table 1, *P* > 0.05). The reads were classified into 1,653 OTUs that were used for downstream analysis. More OTUs were identified in urine from cancer patients, with an average of 120 OTUs in cancer group and 56 OTUs in control group (*P* = 0.008). Higher Observed Species index (richness, *P* = 0.008), Chao1 index (richness, *P* = 0.008), Ace index (richness, *P* = 0.003), Shannon index (diversity, *P* > 0.05) and lower Simpson index (diversity, *P* > 0.05) were presented in cancer group, which indicates that bacterial richness significantly increased in cancer patients while difference of species diversity was not significant (Figures 1A–E, Table 1).

Of interest was that we found that bacterial richness increased in HER group and HEP group, compared to LER group and LEP group, respectively (Supplementary Figures 1A–E, 2A–E; Supplementary Table 4), though no significant association was found between alpha diversity and tumor grade (Supplementary Figures 3A–E; Supplementary Table 5).

**Figure 2:**
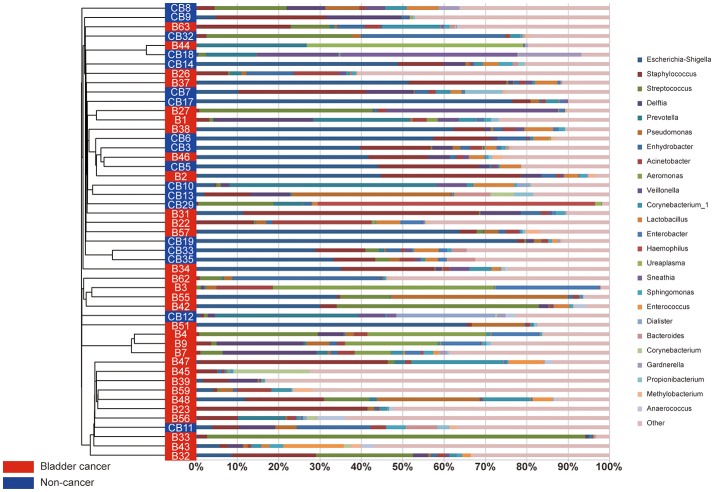
The urinary microbiota profile of participants. A clear hierarchical clustering of cancer samples was observed in the dendrogram at genus level (**left**; based on the unweighted UniFrac distance metric). In the histogram **(right)**, each colored box represents a bacterial taxon and each bar, a subject. The height of a colored box represents the relative abundance of that organism within the sample. Bacterial genera with a relative abundance <0.5% and unclassified genera are grouped as “Other”.

A comparison of urine from cancer patients and that from non-cancer controls showed significantly different bacterial profiles on unweighted UniFrac PCoA plots (Figure 1F). The PERMANOVA performed on the data set showed that the observed differences were statistically significant (*F* = 1.97, *P* < 0.05; *F* = 2.53, *P* < 0.001; and *F* = 1.61, *P* < 0.05, for weighted UniFrac, unweighted UniFrac and Bray Curtis distances, respectively). In addition, a clear hierarchical clustering of cancer samples was observed on dendrogram based on unweighted UniFrac distance metric (Figure 2). We next studied whether the microbial profile was different among different risk level and tumor grade. The results showed that the microbiota composition of patients with high risk of recurrence and progression was significantly different from that of patients with low risk of recurrence and progression (PERMANOA, *F* = 2.31, *P* < 0.01, LER vs. HER; *F* = 2.40, *P* < 0.01, LEP vs. HEP, for weighted UniFrac distances). However, no significant difference in the microbiota profiles of bladder cancer was observed for tumor grade (Supplementary Figures 1F, 2F, 3F, Supplementary Table 6).

### Relative abundance of urinary bacteria in cancer and control samples

**Figure 3:**
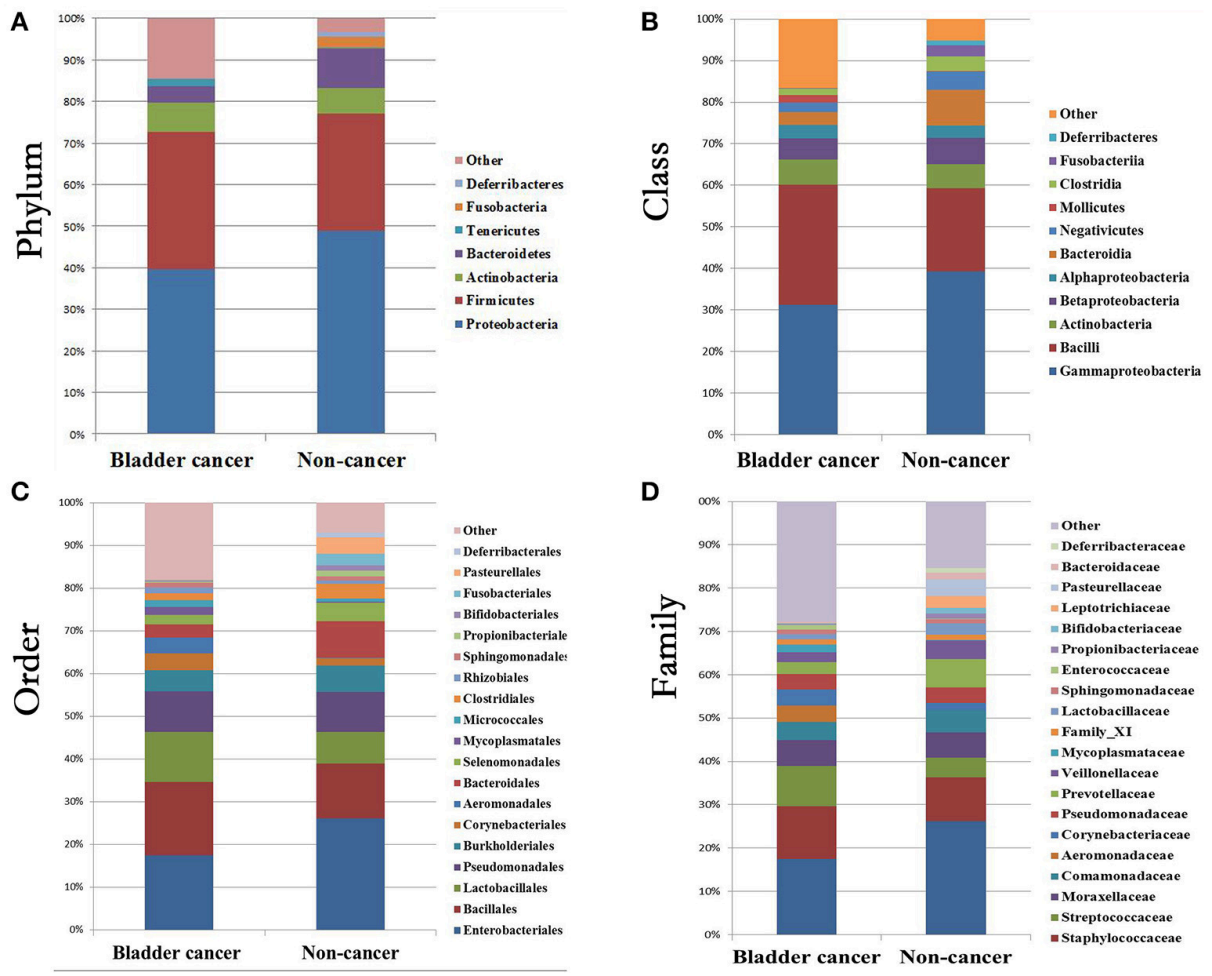
Bacterial average relative abundance in bladder cancer and non-cancer samples. Average distribution of major taxa is represented by bar graphs. **(A)** phylum; **(B)** class; **(C)** order; **(D)** family. Each colored box represents a bacterial taxon and the height of a colored box represents the relative abundance of that organism within the sample. Bacterial genera with a relative abundance <1% and unclassified genera are grouped as “Other”.

At phylum level, the urinary microbiota was dominated by *Proteobacteria* (39.7% cancer, 49.0% control) and *Firmicutes* (32.8% cancer, 28.1% control), followed by *Actinobacteria* (7.0% cancer, 6.2% control) and *Bacteroidetes*(3.9% cancer, 9.4% control) (Figure 3A, Table 2). The microbial composition of all samples at class, order and family level were demonstrated in Figures 3B–D. In addition, the genera compositions of all samples were demonstrated in Figure 2. Though bacterial relative abundance at phylum, class or order level differed between cancer and non-cancer group, no significant difference was found using Metastats algorithm after False Discovery Rate adjustment (Table 2). However, it is notable that *Sphingobacteriaceae* (*P* = 0.047) was significantly more abundant in patients and *Thermoactinomycetaceae* (*P* = 0.005) in control group at family level, while *Acinetobacter* (*P* = 0.048) were significantly more abundant in cancer patients and *Serratia* (*P* = 0.003), *Proteus* (*P* = 0.003), *Laceyella* (*P* = 0.003) in non-cancer group at genus level (Table 2).

**Table 2:** Comparison of relative abundance of urinary microbiome between cancer group and control group at all taxonomic levels.

**Taxa**		**Cancer**	**Non-cancer**	***P*-value**	**FDR**
Phylum	*Proteobacteria*	39.704	48.954	Ns	Ns
	*Firmicutes*	32.840	28.144	Ns	Ns
	*Actinobacteria*	7.073	6.183	Ns	Ns
	*Bacteroidetes*	3.932	9.439	Ns	Ns
Class	*Gammaproteobacteria*	31.218	39.250	Ns	Ns
	*Bacilli*	28.863	20.054	Ns	Ns
	*Actinobacteria*	6.143	5.771	Ns	Ns
	*Betaproteobacteria*	5.061	6.317	Ns	Ns
Order	*Enterobacteriales*	17.435	26.133	Ns	Ns
	*Bacillales*	17.112	12.756	Ns	Ns
	*Lactobacillales*	11.790	7.431	Ns	Ns
	*Corynebacteriales*	3.839	1.733	Ns	Ns
	*Bacteroidales*	3.049	8.619	Ns	Ns
Family	*Enterobacteriaceae*	17.470	26.173	Ns	Ns
	*Staphylococcaceae*	12.213	10.161	Ns	Ns
	*Streptococcaceae*	9.286	4.567	Ns	Ns
	*Thermoactinomycetaceae*	0.000	0.30185	0.001	0.005
	*Sphingobacteriaceae*	0.225	0.012	0.012	0.047
	*Carnobacteriaceae*	0.140	0.022	Ns	Ns
Genus	*Escherichia-Shigella*	15.373	24.460	Ns	Ns
	*Staphylococcus*	12.154	10.123	Ns	Ns
	*Streptococcus*	9.280	4.587	Ns	Ns
	*Aeromonas*	3.782	0.018	0.046	Ns
	*Acinetobacter*	3.252	1.092	0.018	0.048
	*Bacteroides*	0.064	1.584	0.028	Ns
	*Lactobacillus*	1.176	2.626	Ns	Ns
	*Serratia*	0.000	0.984	0.001	0.003
	*Proteus*	0.000	0.312	0.001	0.003
	*Laceyella*	0.000	0.304	0.001	0.003
	*Fusobacterium*	0.049	0.016	Ns	Ns

*Data were reported as mean percentage; FDR, P-value after false discovery rate adjustment; Ns, not significant (based on P < 0.05)*.

### Specific genera associated with bladder cancer

**Figure 4:**
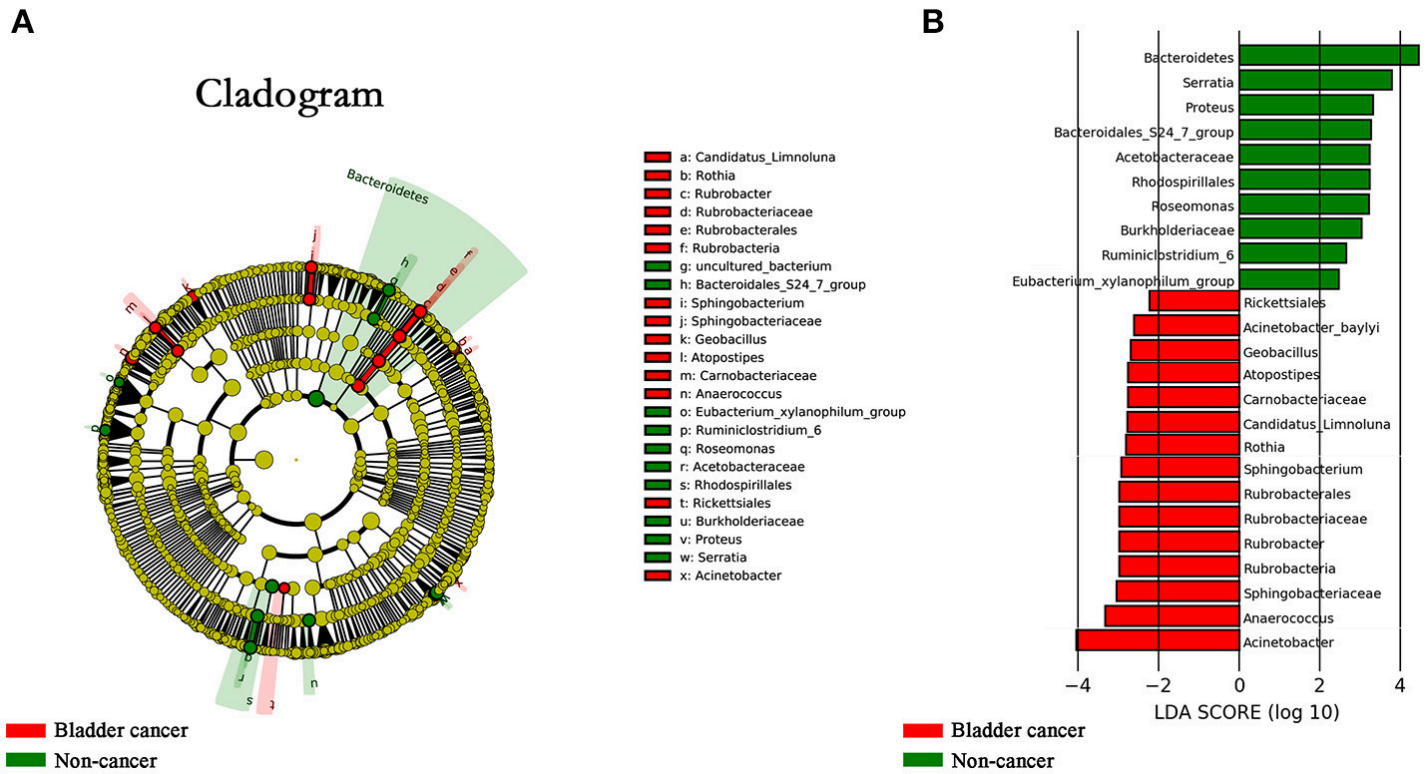
Microbial taxa associated with bladder cancer. **(A)** Cladogram representation of the urinary microbial taxa associated with bladder cancer (red) and non-cancer (green). **(B)** Association of specific microbiota taxa with cancer group and non-cancer group by linear discriminant analysis effect size (LEfSe). Red indicates taxa enriched in cancer group and green indicates taxa enriched in non-cancer group.

The LEfSe, which allows for identifying specific taxa associated with cancer, showed significantly higher compositional abundances of *Acinetobacter, Anaerococcus, Rubrobacter, Sphingobacterium, Atopostipes, Geobacillus* in cancer patients and *Serratia, Proteus, Roseomonas, Ruminiclostridium-6, and Eubacterium–xylanophilum* in control group at genus level (Figures 4A,B). Further analysis showed that *Acinetobacter* (cancer vs. control; 31 of 31 vs. 15 of 18, *P* = 0.044), *Anaerococcus* (cancer vs. control; 19 of 31 vs. 5 of 18, *P* = 0.035), *Rubrobacter* (cancer vs. control; 30 of 31 vs. 11 of 18, *P* = 0.002), *Sphingobacterium* (cancer vs. control; 13 of 31 vs. 2 of 18, *P* = 0.024), *Atopostipes* (13 of 31 vs. 2 of 18, *P* = 0.024) and *Geobacillus* (cancer vs. control; 12 of 31 vs. 1 of 18, *P* = 0.017) were detected in more cancer group samples than control group samples.

Since distinct difference of microbial profile was observed among groups with different risk of recurrence and progression, we next identified the specific taxa associated with high risk of recurrence and progression by using LEfSe analysis. The results showed that 6 genera were overrepresented in patients with high risk of recurrence and 4 genera in patients with high risk of progression, including *Herbaspirillum, Gemella, Bacteroides, Porphyrobacter, Faecalibacterium, Aeromonas* in HER group and *Herbaspirillum, Porphyrobacter, Bacteroides, Marmoricola* in HEP group (Supplementary Figures 4, 5).

### Potential functional pathways associated with bladder cancer

**Figure 5:**
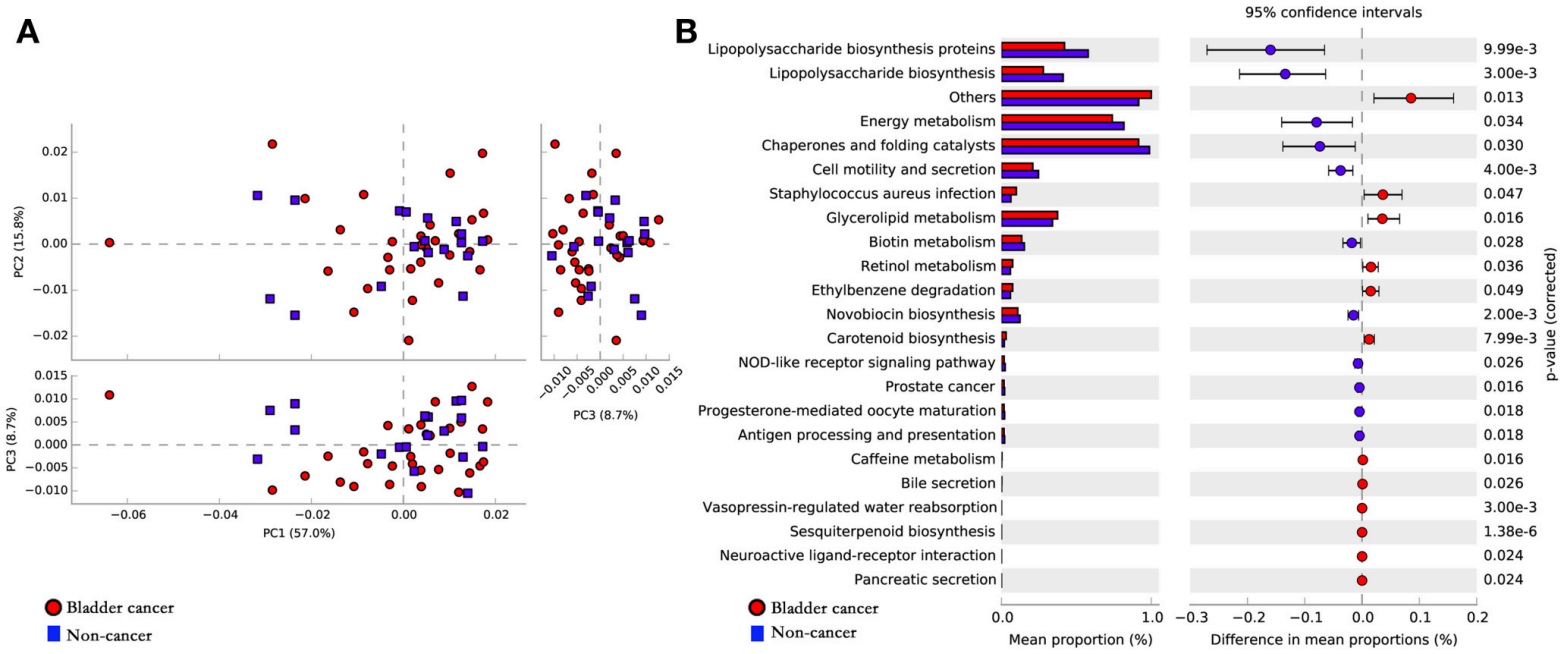
Potential functional pathways associated with bladder cancer. **(A)** Principal component analysis (PCA) plot comparing the metagenome predictions (for KEGG orthology using PICRUSt) of the microbiota of patients with bladder cancer and non-cancer. **(B)** Microbial pathways that were significantly differentially enriched between the cases and controls. Red indicates taxa enriched in cancer group and blue indicates taxa enriched in non-cancer group.

To infer the functional pathways based on the microbial community profiles we utilized PICRUSt. Overall, the microbial profiles present in patients with bladder cancer and non-cancer could not be distinguished clearly based on their functions (Figure 5A). The predicted KEGG pathways significantly enriched in bladder cancer included *Staphylococcus aureus* infection, glycerolipid metabolism, retinol metabolism, ethylbenzene degradation and carotenoid biosynthesis (Figure 5B).

## Discussion

In this study, we have characterized the urinary microbial profile of bladder cancer by using 16S rRNA gene sequencing and the results showed that bacterial richness significantly increased in bladder cancer patients. In addition, higher bacterial richness was presented in urine samples with higher risk of recurrence and progression, which suggests that higher bacterial richness may be a potential indicator of high risk of recurrence and progression of NMIBC.

A reduction in microbial diversity has now been considered as a feature of gut disease, such as ulcerative colitis, Crohn's disease and colorectal cancer (Lepage et al., 2011; Ahn et al., 2013; Gevers et al., 2014). However, no consistent changes in microbial diversity were found among urinary tract disorders. Increased microbial diversity was observed in urgency urinary incontinence (Pearce et al., 2015), reduced diversity was found in interstitial cystitis (Siddiqui et al., 2012) and overactive bladder (Wu et al., 2017), while no significant difference in microbial diversity was found in prostate cancer (Shrestha et al., 2018).

Similar to microbiome inhabiting the gut, inter-individual microbial community heterogeneity of the urinary tract is influenced by lots of genetic and environmental factors, such as spatial distribution or lifestyle. However, despite the distinct inter-individual differences between samples, a common microbial feature appears to emerge, as shown in the PCoA analysis that clustered cancer group and control group separately (Figure 1F), indicating a possibility on common dysbiosis associated to bladder cancer. Studies suggest that changes in microbial composition and function may contribute to carcinogenesis, tumor progression and dissemination at these sites (Schwabe and Jobin, 2013). Alteration of urinary microbiome might be also involved in the development and progression of bladder cancer.

Bucevic Popovic et al. reported that no significant difference was observed in overall microbial profiles between bladder cancer (*n* = 12) and control group (*n* = 11) (Bucevic Popovic et al., 2017). Number of cases, ethnicity, gender and age may be reasons of different results between our study and Bucevic Popovic's study.

*Acinetobacter* and *Anaerococcus* were two genera found in higher abundances in bladder cancer patients than in non-cancer group. *Acinetobacter* spp. was reported one of the most abundant Gram-negative bacteria isolated from the urine of cattle affected by urothelial tumors of the urinary bladder (Roperto et al., 2012). *Acinetobacter* is a complex genus associated with nosocomial infections, including urinary tract infections. The virulence factors of *Acinetobacter baumannii*, a species of *Acinetobacter*, identified to date were confirmed to be involved in biofilm formation, adherence and invasion of epithelial cells, bacterial dissemination by degrading phospholipids present at mucosal barrier, and escape from the host immune response (McConnell et al., 2013). As for *Anaerococcus*, it was reported as a member of the Gram-positive anaerobic cocci, which was able to induce inflammation, remodeling of extracellular matrix (ECM) and re-epithelialization (Murphy and Frick, 2013). Based on above analysis, we raise an intriguing possibility that the interplay of ECM and microbiome and concomitant inflammation might play a role in bladder carcinogenesis.

The link between chronic inflammation, microbiome and the initiation and progression of solid tumors has been established for various neoplastic diseases, especially colorectal cancer (Irrazábal et al., 2014). Transient inflammation is considered as part of body's immune defense against pathogen, but persistent inflammation could potentially contribute to the development of cancer (Ainsworth, 2017). One large epidemiological study reported that repeated, regular bouts of cystitis were associated with increased risk of bladder cancer (Vermeulen et al., 2015). Alfano et al. reported that bacteria produce proteases, acting as virulence factors with crucial roles in host tissue degradation, as well as immune system evasion and destruction of host physical barriers, further promoting inflammation, remodeling of ECM and the generation of oxygen radicals, which results in mutagenesis that may promote the onset and progression of cancer (Alfano et al., 2016).

In our study, the LEfSe analysis showed that bacterial taxa along *Sphingobacteriaceae*-to-*Sphingobacterium* lineage (Figures 4A,B) were enriched in cancer patients. *Sphingobacterium* are recognized as etiological agents of cystic fibrosis and urinary tract infections. Besides, ceramides and sphingophospholipids of *S. spiritivorum*, one species of the genus *Sphingobacterium*, could induce DNA fragmentation, caspase-3 activation, changes in morphology and cell cycle shortening (Lambiase, 2014). The PICRUSt showed that in comparison with controls, *Staphylococcus aureus* infection was increased in patients with bladder cancer. *Staphylococcus aureus* could produce various enzymes such as alkaline protease, elastase and phospholipase C, which could degrade ECM components, break down elastin, disrupt tight junction, damage tissue, and cleaves various bonds in phospholipids. Above analysis also raises the possibility that microbiome-mediated modifications of ECM and concomitant inflammation might play an role in the initiation and development of bladder cancer. However, this study cannot answer the question whether changes in the microbiome contribute to cancer or vice versa. Numerous studies are required to determine whether the described profile is associated to, correlated with, or even responsible for bladder carcinogenesis and development.

Xu et al. reported enrichment of *Streptococcus spp*. in urine from urothelial carcinoma patients (*n* = 8) compared to healthy individuals (*n* = 6) (Xu et al., 2014). Bucevic Popovic et al. reported that genus *Fusobacterium* was significantly enriched in urine of bladder cancer patients. *Streptococcus* and *Fusobacterium* were also detected in our study. However, despite relative abundance of both genera was higher in bladder cancer patients, no significant difference was found between cancer and non-cancer group using Metastats algorithm after False Discovery Rate adjustment (Table 2).

Alfano et al. reported that specific pathogenic bacteria might promote the initiation and development of malignancies and tumor-associated microenvironments probably selectively facilitate the growth of specific bacteria in turn (Alfano et al., 2016). Enrichment of *Herbaspirillum, Porphyrobacter*, and *Bacteroides* was observed in bladder cancer patients with high risk of recurrence and progression (Supplementary Figures 4, 5), which suggests that these genera maybe potential biomarkers for risk stratification and to predict cancer prognosis. In addition, given the promising results of fecal microbiota transplantation, which was used to “re-establish the balance of nature” within the intestinal environment for the treatment of several refractory gastrointestinal disorders, strategies to restore normal bladder-associated microenvironment might be a potential option to reduce incidence or recurrence of bladder cancer.

Immune checkpoint targets have become the focus of investigation for the treatment of bladder cancer, including programmed death ligand-1 (PD-L1). Atezolizumab was the first PD-L1 inhibitor confirmed active in bladder cancer and is currently the only PD-L1 inhibitor approved by the FDA for patients with metastatic or locally advanced urothelial carcinoma based on promising overall response rates in clinical trials (Bellmunt et al., 2017). Though checkpoint blockade therapies have had remarkable results in management for bladder cancer, only small part of patients responds to PD-L1 blockers (Inman et al., 2017). It has become evident that microbiome plays a crucial role in modulating the response to cancer therapy, including immunotherapy (Roy and Trinchieri, 2017). Zitvogel *et al* reported that the microbiome could affect the therapeutic efficacy of PD-L1 blockade (Zitvogel et al., 2016). If the true association between microbial community and bladder carcinogenesis is confirmed in the future, urinary microbiome might be a target for enhancing bladder cancer responses PD-L1 inhibitor.

Since gender can affect microbiome studies (Kim et al., 2017) and men are at considerably higher risk of developing bladder cancer, this study included only male patients (Dobruch et al., 2016). On one hand the restriction to male patients limited the generalizability of the data, on the other hand the restriction minimized confounding factor of gender. In addition, to avoid potential bias caused by hospital noise and environmental changes, we recruited non-neoplastic patients as controls. However, urinary microbiota may be influenced by various disorders, despite excluding confounding factors of neoplasms.

Our study is not devoid of limitations. Firstly, it is not possible to determine the cause-effect relationship between microbiome and bladder cancer for retrospective study and low number of cases. Thus, prospective follow-up studies with a larger sample number and animal experiment studies will be needed to clarify the role of microbiome in development and progression of bladder cancer. Secondly, despite the fact that potential harm brought by catheterization can be avoided, mid-stream urine sample collected by the clean catch method is potentially contaminated with microbiota surrounding the urethral orifice. Thirdly, bladder mucosa would provide the best samples to characterize the bladder-associated microbiome, which could rule out sample contamination with microorganisms present in the urethra. However, obtaining bladder biopsies or suprapubic aspirates in healthy individuals is unethical. Hence, profiling microbiome of tumor, peri-tumor and non-tumor tissues of bladder cancer patients would be crucial to ascertain the association between pathogenic effect of microbial dysbiosis and the onset, progression and relapse of cancer. Another limitation is that we did not have relevant data on urinary incontinence or retention profiles. The effect of profiles on urination may contribute to the results in the study. Last but not least, the 16S rRNA gene sequencing-based approach enabled us to detect bacteria that present even in low numbers on one hand, on the other hand this method cannot identify bacteria well at species level and detect non-bacterial microorganisms, such as viruses and fungi (Ainsworth, 2017).

In conclusion, we have profiled the urinary microbiome associated with bladder cancer in the most comprehensive study to date. Our study suggests that urinary microbiota may be associated with bladder cancer, but the cause-effect relationship remains unclear. When a true association between urinary microbiota and bladder cancer is determined in the future, a better understanding of the role of microbiome in the development and progression of bladder cancer could provide novel diagnostic and prognostic biomarkers, as well as more microbiome-targeted therapeutic options.

## Author contributions

PW, GZ, JZ: conception and design; GZ, YC, JC: acquisition of data; GZ, YC, JZ: analysis and interpretation of data; PW, GZ, JZ: drafting of the manuscript; PW, WH, JRZ: critical revision of the manuscript for important intellectual content; PW, GZ, JZ, JLZ: statistical analysis; PW: obtaining funding; PW: supervision.

### Conflict of interest statement

The authors declare that the research was conducted in the absence of any commercial or financial relationships that could be construed as a potential conflict of interest.
