# Corrigendum: Profiling the Urinary Microbiota in Male Patients With Bladder Cancer in China

**DOI:** 10.3389/fcimb.2018.00429

**Published:** 2018-12-11

**Authors:** Peng Wu, Guihao Zhang, Jie Zhao, Jiawei Chen, Yang Chen, Weina Huang, Jialei Zhong, Jiarong Zeng

**Affiliations:** ^1^Department of Urology, Nanfang Hospital, Southern Medical University, Guangzhou, China; ^2^School of Pharmaceutical Sciences, Southern Medical University, Guangzhou, China

**Keywords:** urinary bladder neoplasms, urinary tract, microbiota, extracellular matrix, inflammation

In the original article, there was an error. Readers were unable to access the 16S sequencing data submitted to the Short Read Archive (SRA) under the accession number SUB3915640.

A correction has been made to **MATERIALS AND METHODS, DNA Isolation and 16S rRNA Gene Sequencing**:

“To avoid contamination, DNA isolation was performed using the cultured cells protocol supplied with the DNeasy Blood and Tissue Kit (Qiagen, Germany) in a laminar flow hood. The concentration of extracted DNA was determined through a Nanodrop ND-1000 spectrophotometer (Thermo Electron Corporation, USA). The genomic DNA isolated from the clinical samples was amplified using primer sets specific for V4 regions (515F: GTGCCAGCMGCCGCGGTAA; and 806R:GGACTACHVGGGTWTCTAAT). In order to evaluate contribution of extraneous DNA from reagents, extraction negative controls (no urine) and PCR negative controls (no template) were included. The resultant PCR products were purified by Qiaquick PCR purification kit (Qiagen, Valencia, CA). Finally, purified samples were normalized to equal DNA concentration and sequenced using the Illumina Miseq sequencer (Illumina, Inc., USA). The 16S rRNA gene sequences have been submitted to the Short Read Archive (SRA) under accession number PRJNA486651”.

The authors apologize for this error and state that this does not change the scientific conclusions of the article in any way. The original article has been updated.

