# Supplementary figures and images for: Profiling the Urinary Microbiota in Male Patients With Bladder Cancer in China
Source: Front Cell Infect Microbiol. 2018 May 31;8:167. doi: 10.3389/fcimb.2018.00167 (PMC5990618; doi:10.3389/fcimb.2018.00167)

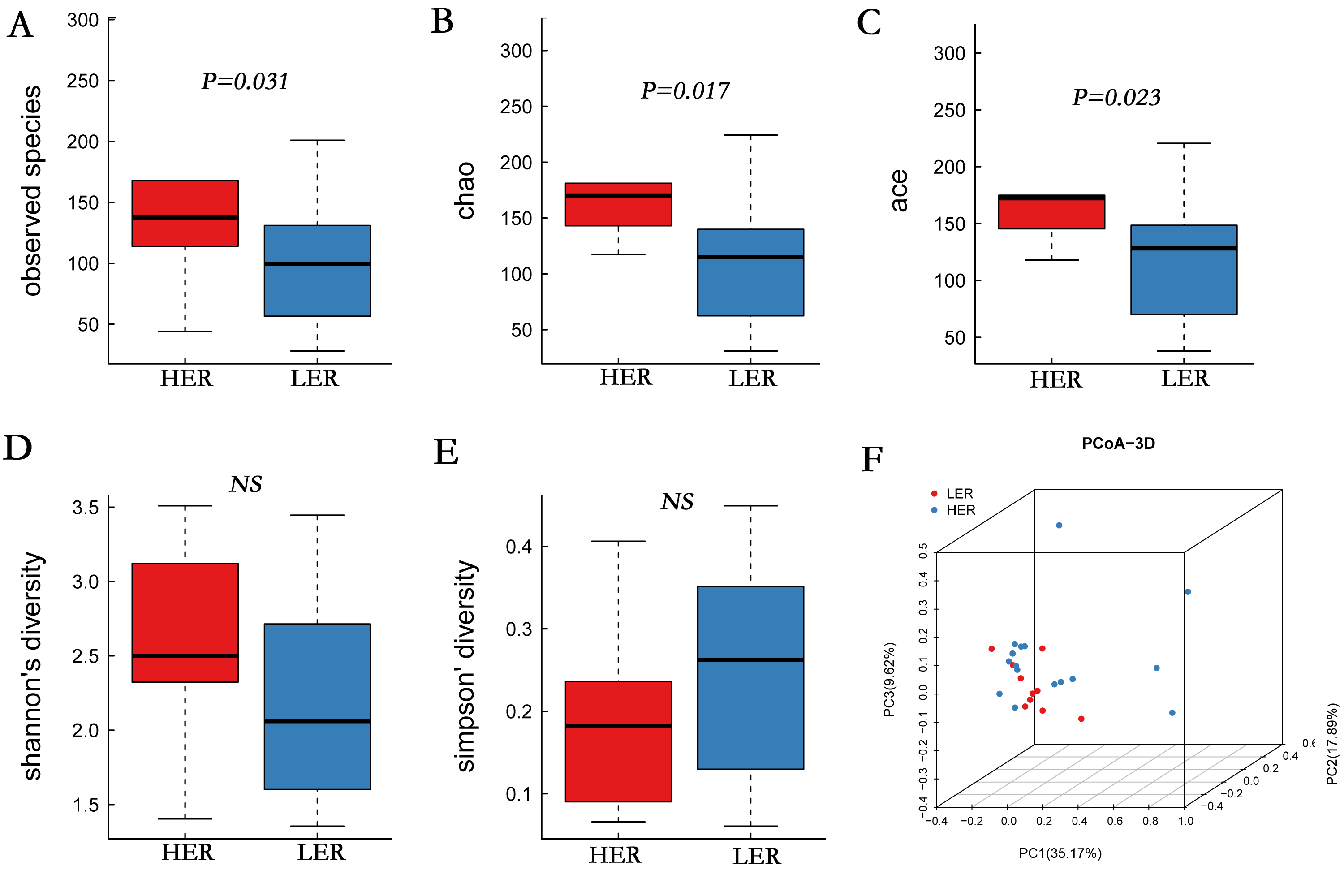

Supplement: Supplementary file 1 [file Image_1.TIF]

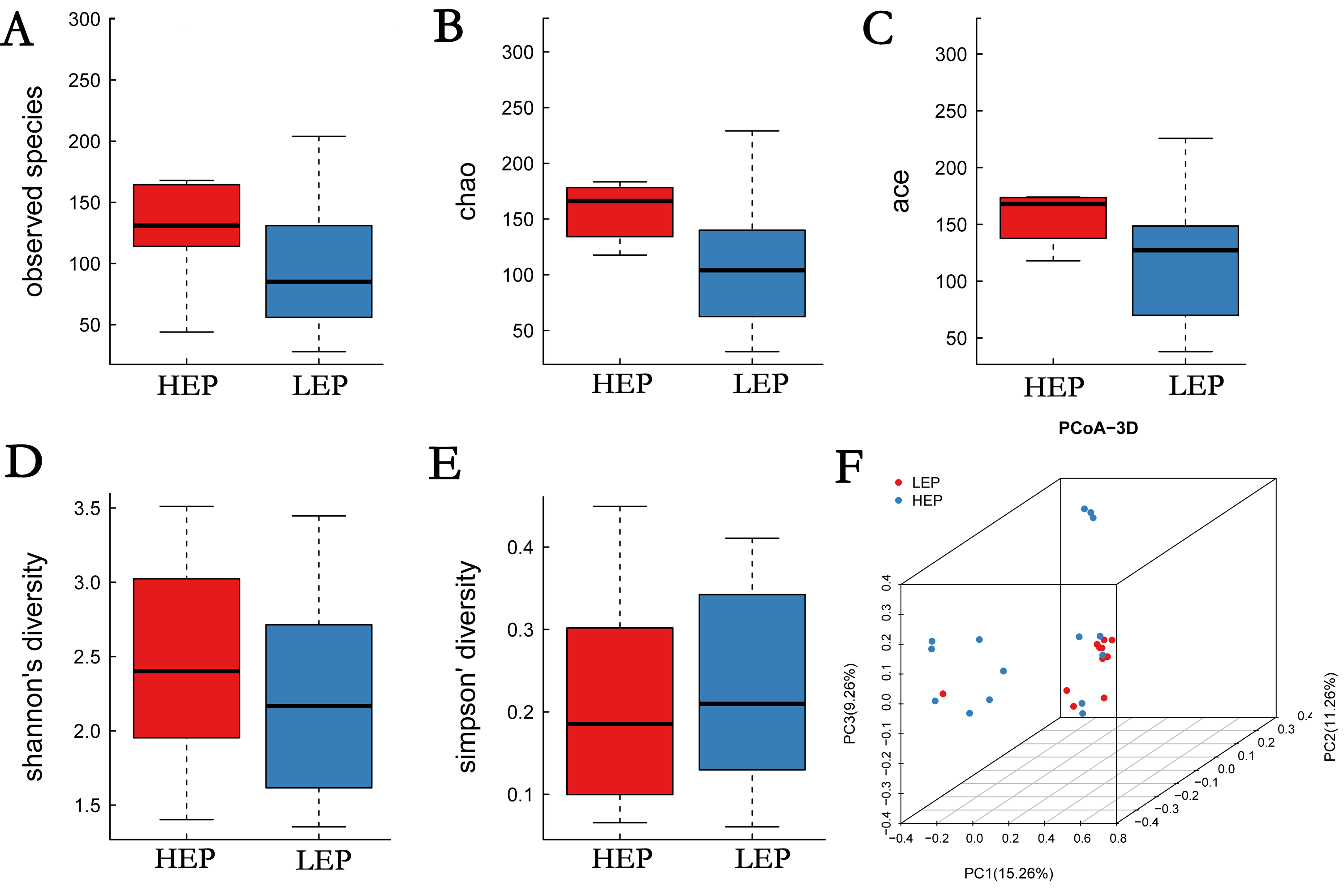

Supplement: Supplementary file 2 [file Image_2.TIF]

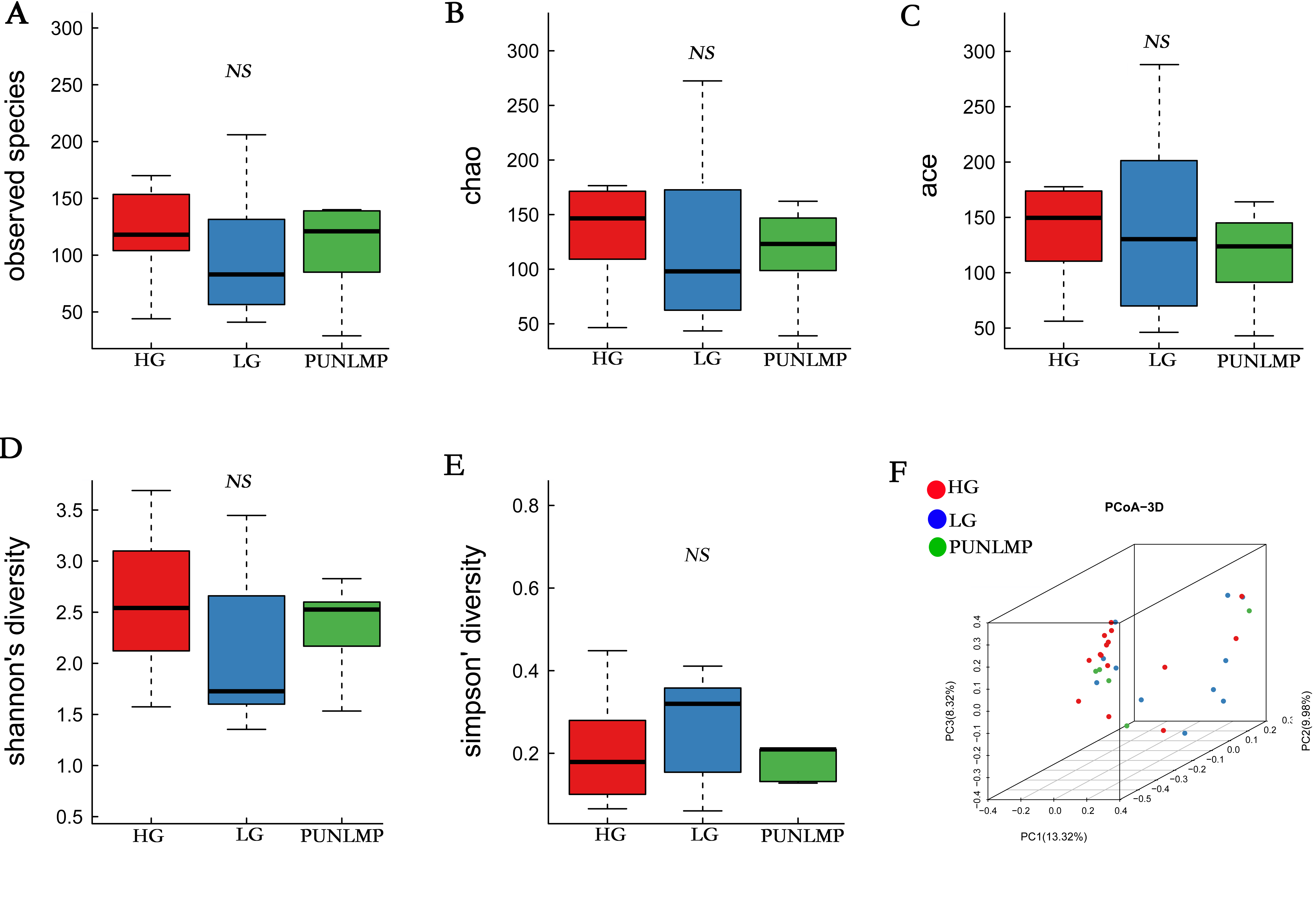

Supplement: Supplementary file 3 [file Image_3.TIF]

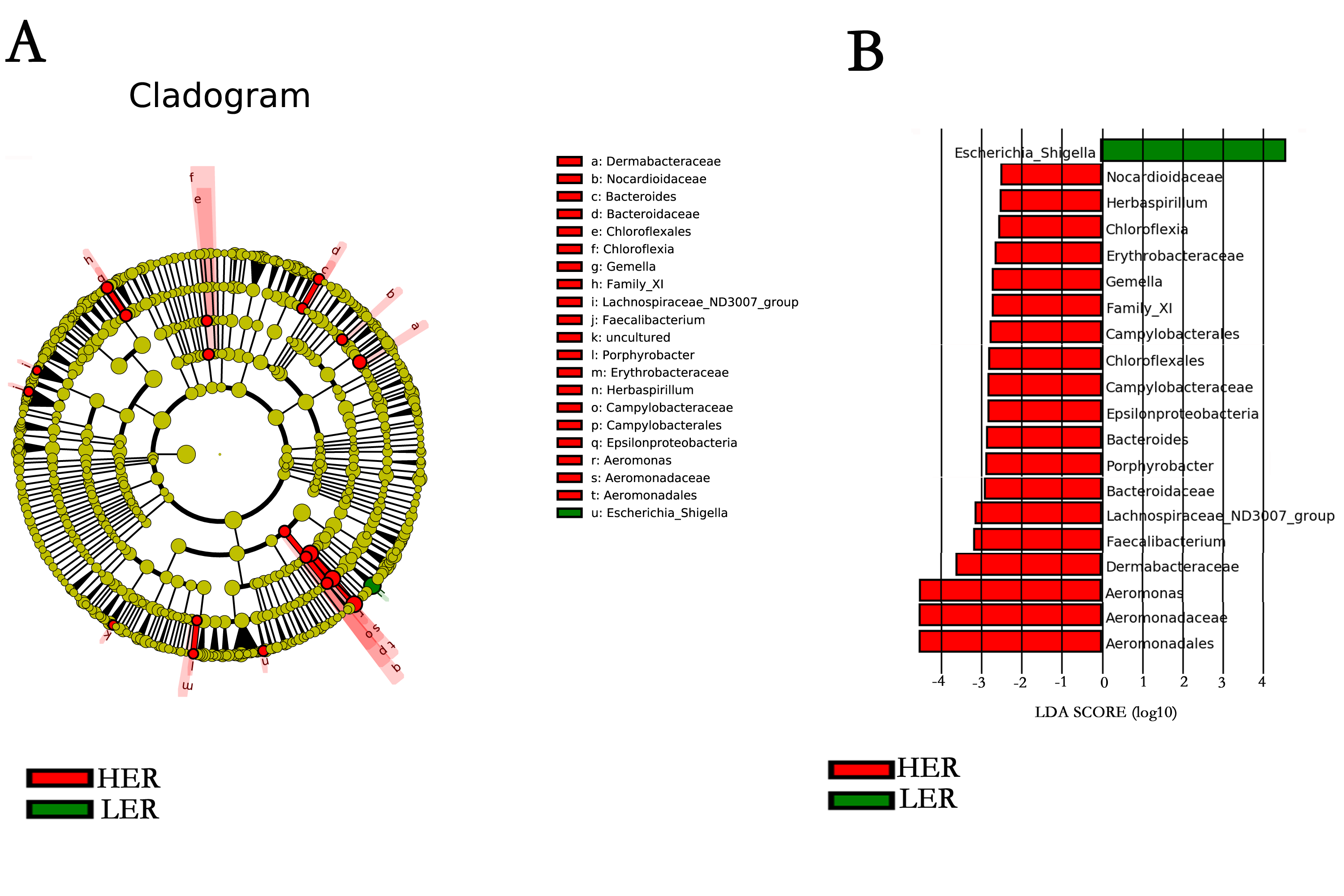

Supplement: Supplementary file 4 [file Image_4.TIF]

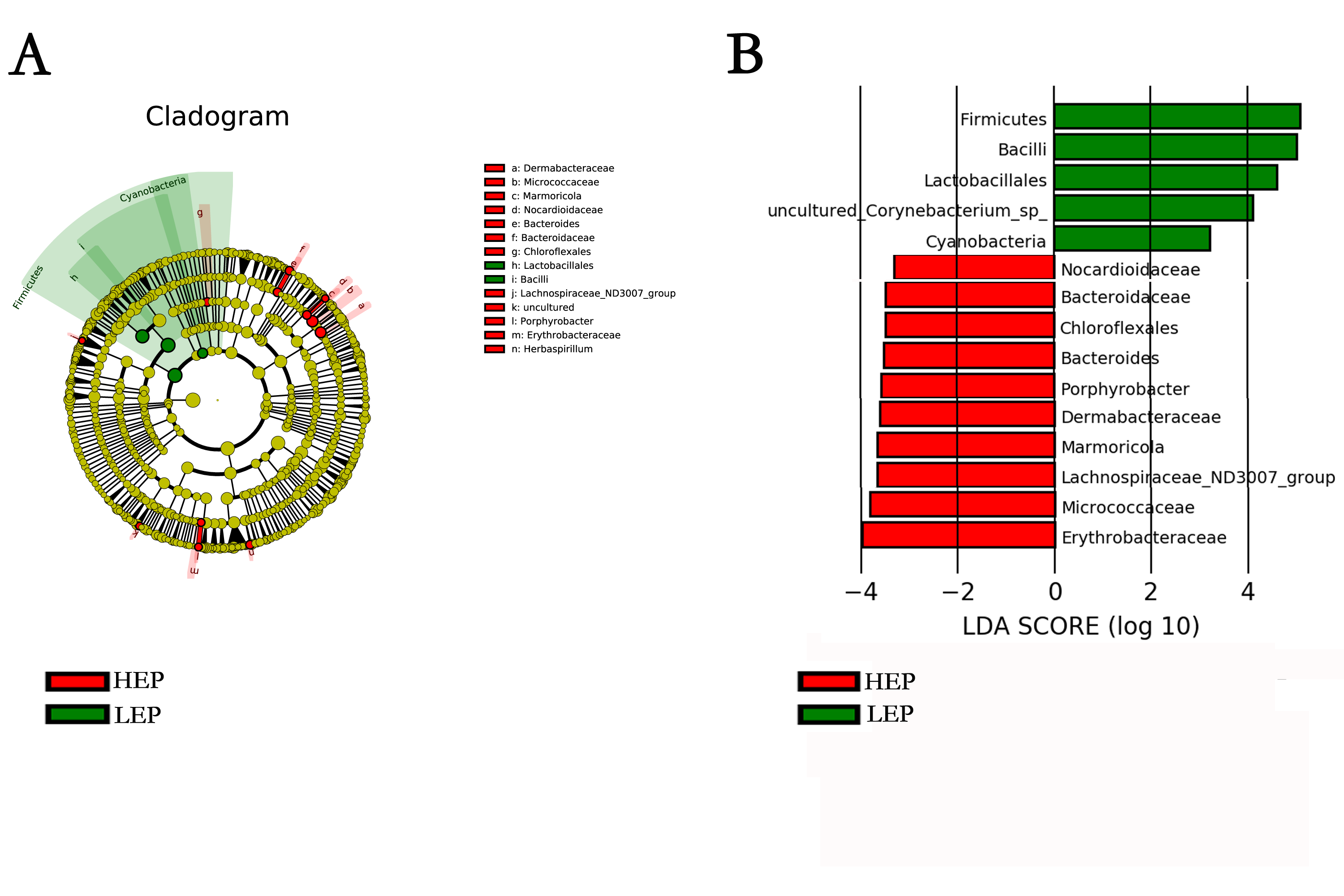

Supplement: Supplementary file 5 [file Image_5.TIF]
