# Supplementary material for: Profiling the Urinary Microbiota in Male Patients With Bladder Cancer in China
Source: Front Cell Infect Microbiol. 2018 May 31;8:167. doi: 10.3389/fcimb.2018.00167 (PMC5990618; doi:10.3389/fcimb.2018.00167)
Supplement: Supplementary file 6 [file Image_6.PDF]

LEGEND TO SUPPLEMENTARY FIGURE

Supplementary Figure 1. Alpha and principal coordinate analysis (PCoA) for LER (recurrence score of EORTC≤4) and HER (recurrence score of EORTC≥5) samples. (A)-(E).Box-plot showing alpha diversity in samples using different metrics (A. observed species; B. Chao1 ; C. Ace; D. Shannon index; E. Simpson index); (F). PCoA plots of weighed UniFrac distances in which samples were coloured by clinical outcome. The PERMANOVA performed on the weighted UniFrac distances showed that the observed differences were statistically significant (999 permutations; F=2.31; $P<0.01$); NS, not significant.

Supplementary Figure 2. Alpha and principal coordinate analysis (PCoA) for LEP (progression score of EORTC≤6) and HEP (progression score of EORTC≥7) samples. (A)-(E).Box-plot showing alpha diversity in samples using different metrics (A. observed species; B. Chao1; C. Ace; D. Shannon index; E. Simpson index); (F). PCoA plots of unweighed UniFrac distances in which samples were coloured by clinical outcome. The PERMANOVA performed on the unweighted UniFrac distances showed that the observed differences were statistically significant (999 permutations; F=2.05; $P<0.01$); NS, not significant.

Supplementary Figure 3. Alpha and principal coordinate analysis (PCoA) for PUNLMP (papillary urothelial neoplasm of low malignant potential); LG (low-grade papillary urothelial carcinoma) and HG (high-grade papillary urothelial carcinoma) samples. (A)-(E).Box-plot showing alpha diversity in samples using different metrics (A. observed species; B. Chao1; C. Ace; D. Shannon index; E. Simpson index); (F). PCoA plots of unweighed UniFrac distances in which samples were coloured by clinical outcome. The PERMANOVA performed on the unweighted UniFrac distances showed that the observed differences were not significant (999 permutations; F=0.90; $P>0.05$); NS, not significant.

Supplementary Figure 4. Microbial taxa associated with bladder cancer. (A) Cladogram representation of the urinary microbial taxa associated with high risk of recurrence (HER, red) and low risk of recurrence (LER, green). (B) Association of specific microbiota taxa with HER and LER group by linear discriminant analysis effect size (LEfSe). Red indicates taxa enriched in HER group and green indicates taxa enriched in LER group.

Supplementary Figure 5. Microbial taxa associated with bladder cancer. (A) Cladogram representation of the urinary microbial taxa associated with high risk of progression (HEP, red) and low risk of progression (LEP, green). (B) Association of specific microbiota taxa with HEP and LEP group by linear discriminant analysis effect size (LEfSe). Red indicates taxa enriched in HEP group and green indicates taxa enriched in LEP group.
