# Supplementary material for: Profiling the Urinary Microbiota in Male Patients With Bladder Cancer in China
Source: Front Cell Infect Microbiol. 2018 May 31;8:167. doi: 10.3389/fcimb.2018.00167 (PMC5990618; doi:10.3389/fcimb.2018.00167)
Supplement: Supplementary file 8 [file Table_2.DOCX]

| **Supplementary Table 2\|Distribution of patients with bladder cancer** | | |  |
| --- | --- | --- | --- |
| **Characteristic** | **Num. of cases** | **(%) of cases** | |
| **Age (y)** |  |  | |
| <50 | 8 | 25.8 | |
| 50~59 | 4 | 12.9 | |
| 60~69 | 12 | 38.7 | |
| ≥70 | 7 | 22.6 | |
| **Smoking index** |  |  | |
| 0 | 8 | 25.8 | |
| <400 | 4 | 12.9 | |
| ≥400 | 19 | 61.3 | |
| **Tumour grading^1^** |  |  | |
| PUNLMP | 5 | 16.1 | |
| Low grade | 11 | 35.5 | |
| High grade | 10 | 48.4 | |
| **EORTC-Recurrence score^2^** |  |  | |
| 0 | 5 | 19.2 | |
| 1~4 | 11 | 42.3 | |
| 5~9 | 8 | 30.8 | |
| 10~17 | 2 | 7.7 | |
| **EORTC-Progression score^2^** |  |  | |
| 0 | 9 | 34.6 | |
| 2~6 | 6 | 23.1 | |
| 7~13 | 8 | 30.8 | |
| 14~23 | 3 | 11.5 | |
| 1. according to the 2004 WHO grading system; 2. according to European Organization for Research and Treatment of Cancer (EORTC) scoring system and tables | | | |
