# Supplementary material for: Profiling the Urinary Microbiota in Male Patients With Bladder Cancer in China
Source: Front Cell Infect Microbiol. 2018 May 31;8:167. doi: 10.3389/fcimb.2018.00167 (PMC5990618; doi:10.3389/fcimb.2018.00167)
Supplement: Supplementary file 9 [file Table_3.DOCX]

| **Supplementary Table 3.1\| Weighting used to calculate disease recurrence and progression scores of European Organization for Research and Treatment of Cancer (EORTC)** | | | | | | |
| --- | --- | --- | --- | --- | --- | --- |
| **Factor** | | **Recurrence** | | | **Progression** | |
| Number of tumors | | | | | | |
| Single | | 0 | | | 0 | |
| 2-7 | | 3 | | | 3 | |
| ≥8 | | 6 | | | 3 | |
| Tumor diameter | | | | | | |
| ＜3cm | | 0 | | | 0 | |
| ≥3 | | 3 | | | 3 | |
| Prior recurrence rate | | | | | | |
| Primary | | 0 | | | 0 | |
| ≤1 recurrence/year | | 2 | | | 2 | |
| > 1 recurrence/year | | 4 | | | 2 | |
| Category | | | | | | |
| Ta | | 0 | | | 0 | |
| T1 | | 1 | | | 4 | |
| Concurrent CIS | | | | | | |
| No | | 0 | | | 0 | |
| Yes | | 1 | | | 6 | |
| Grade | | | | | | |
| G1 | | 0 | | | 0 | |
| G2 | | 1 | | | 0 | |
| G3 | | 2 | | | 5 | |
| **Total Score** | | 0-17 | | | 0-23 | |
| **Supplementary Table 3.2\| Probability of recurrence and disease progression according to total score** | | | | | | |
| **Recurrence score** | **Probability of recurrence at 1 year** | | | **Probability of recurrence at 5 years** | | |
|  | % | | (95% CI) | % | | (95% CI) |
| 0 | 15 | | (10-19) | 31 | | (24-37) |
| 1-4 | 24 | | (21-26) | 46 | | (42-49) |
| 5-9 | 38 | | (35-41) | 62 | | (58-65) |
| 10-17 | 61 | | (55-67) | 78 | | (73-84) |
|  | | | | | | |
| **Progression score** | **Probability of progression at 1 year** | | | **Probability of progression at 5 years** | | |
|  | % | | (95% CI) | % | | (95% CI) |
| 0 | 0.2 | | (0-0.7) | 0.8 | | (0-1.7) |
| 2-6 | 1 | | (0.4-1.6) | 6 | | (5-8) |
| 7-13 | 5 | | (4-7) | 17 | | (14-20) |
| 14-23 | 17 | | (10-24) | 45 | | (35-55) |
