# Supplementary material for: Profiling the Urinary Microbiota in Male Patients With Bladder Cancer in China
Source: Front Cell Infect Microbiol. 2018 May 31;8:167. doi: 10.3389/fcimb.2018.00167 (PMC5990618; doi:10.3389/fcimb.2018.00167)
Supplement: Supplementary file 10 [file Table_4.DOCX]

| **Supplementary Table 4.1\| Comparison of richness and diversity estimation in urine samples between LER group and HER group** | | | |
| --- | --- | --- | --- |
|  | **LER**  **(n=16)** | **HER**  **(n=10)** | ***P* Value** |
| **Parameter** |  |  |  |
| Number of OTUs | 102.0(58.3,138.5) | 147.5(117.3,205.3) | *0.031* |
| Chao1 | 116.8(69.1,154.3) | 174.9(141.5,240.1) | *0.017* |
| Ace | 126.1(69.1,152.9) | 177.4(146.3,236.6) | *0.023* |
| Shannon | 2.1(1.6,2.7) | 2.5(2.1,3.2) | *Ns* |
| Simpson | 0.3(0.1,0.4) | 0.2(0.1,0.3) | *Ns* |
| Data were presented as median (first quartile to the third quartile); LER, recurrence score of EORTC≤4; HER, recurrence score of EORTC≥5; OTUs, operational taxonomic units; Ns, not significant. | | | |

| **Supplementary Table 4.2\|Comparison of richness and diversity estimation in urine samples between LEP group and HEP group** | | | |
| --- | --- | --- | --- |
|  | **LEP**  **(n=15)** | **HEP**  **(n=11)** | ***P* Value** |
| **Parameter** |  |  |  |
| Number of OTUs | 85.0(57.0,141.0) | 143.0(119.0,173.0) | *0.041* |
| Chao1 | 106.0(69.0,156.8) | 171.3(134.0,214.6) | *0.024* |
| Ace | 124.1(65.0,154.4) | 174.8(139.6,207.4) | *0.027* |
| Shannon | 2.1(1.6,2.8) | 2.4(1.6,3.1) | *Ns* |
| Simpson | 0.2(0.1,0.4) | 0.2(0.1,0.4) | *Ns* |
| Data were presented as median (first quartile to the third quartile); LEP, recurrence score of EORTC≤6; HEP, recurrence score of EORTC≥7; OTUs, operational taxonomic units; Ns, not significant. | | | |
