# Supplementary material for: Profiling the Urinary Microbiota in Male Patients With Bladder Cancer in China
Source: Front Cell Infect Microbiol. 2018 May 31;8:167. doi: 10.3389/fcimb.2018.00167 (PMC5990618; doi:10.3389/fcimb.2018.00167)
Supplement: Supplementary file 11 [file Table_5.DOCX]

| **Supplementary Table 5\| Comparison of richness and diversity estimation in urine samples among different histological group (PUNLMP vs LG vs HG)** | | | | |
| --- | --- | --- | --- | --- |
|  | **PUNLMP**  **n=5** | **Low grade**  **n=11** | **High grade**  **n=15** | ***P* Value** |
| **Parameter** |  |  |  |  |
| Number of OTUs | 124.0(56.5,146.0) | 85.0(57.0,143.0) | 146.1(117.2,165.5) | *Ns* |
| Chao1 | 127.5(65.1,164.6) | 106.0(69.0,214.6) | 149.6(130.3,178.8) | *Ns* |
| Ace | 128.1(63.4,163.8) | 124.0(65.0,229.0) | 155.2(134.6,181.1) | *Ns* |
| Shannon | 2.5(1.8,2.7) | 1.7(1.5,2.9) | 2.5(2.1,3.2) | *Ns* |
| Simpson | 0.2(0.1,0.3) | 0.3(0.1,0.4) | 0.2(0.1,0.3) | *Ns* |
| Data were presented as median (first quartile to the third quartile); OTUs, operational taxonomic units; Ns, not significant. | | | | |
