# Supplementary material for: Profiling the Urinary Microbiota in Male Patients With Bladder Cancer in China
Source: Front Cell Infect Microbiol. 2018 May 31;8:167. doi: 10.3389/fcimb.2018.00167 (PMC5990618; doi:10.3389/fcimb.2018.00167)
Supplement: Supplementary file 12 [file Table_6.DOCX]

| **Supplementary Table 6.1\|** **PERMANOA based on weighted UniFrac metric (LER vs HER)** | | | | | | | |
| --- | --- | --- | --- | --- | --- | --- | --- |
|  | **DF** | **SumsOfSqs** | **MeanSqs** | **F.Model** | **R2** | **Pr(>F)** | **Significance** |
|  | 1 | 0.3933 | 0.39333 | 2.3051 | 0.08763 | 0.035 | * |
| Residuals | 24 | 4.0953 | 0.17064 | - | 0.91237 | - | - |
| Total | 25 | 4.4886 | - | - | 1.00000 | - | - |
| Significance codes: 0 “***”, 0.001 “**”, 0.01 “*”, 0.05 “.”, 0.1 “”; LER, recurrence score of EORTC≤4; HER, recurrence score of EORTC≥5. | | | | | | | |
| **Supplementary Table 6.2\|** **PERMANOA based on unweighted UniFrac metric (LER vs HER)** | | | | | | | |
|  | **DF** | **SumsOfSqs** | **MeanSqs** | **F.Model** | **R2** | **Pr(>F)** | **Significance** |
|  | 1 | 0.3887 | 0.38866 | 1.1354 | 0.04517 | 0.239 | - |
| Residuals | 24 | 8.2152 | 0.34230 | - | 0.95483 | - | - |
| Total | 25 | 8.6038 | - | - | 1.00000 | - | - |
| Significance codes: 0 “***”, 0.001 “**”, 0.01 “*”, 0.05 “.”, 0.1 “”; LER, recurrence score of EORTC≤4; HER, recurrence score of EORTC≥5. | | | | | | | |
| **Supplementary Table 6.3\|** **PERMANOA based on Bray-Curtis metric (LER vs HER)** | | | | | | | |
|  | **DF** | **SumsOfSqs** | **MeanSqs** | **F.Model** | **R2** | **Pr(>F)** | **Significance** |
|  | 1 | 0.5318 | 0.53175 | 1.5487 | 0.06062 | 0.093 | **.** |
| Residuals | 24 | 8.2405 | 0.34335 | - | 0.93938 | - | - |
| Total | 25 | 8.7723 | - | - | 1.00000 | - | - |
| Significance codes: 0 “***”, 0.001 “**”, 0.01 “*”, 0.05 “.”, 0.1 “”; LER, recurrence score of EORTC≤4; HER, recurrence score of EORTC≥5. | | | | | | | |
| **Supplementary Table 6.4\| PERMANOA based on weighted UniFrac metric (LEP vs HEP)** | | | | | | | |
|  | **DF** | **SumsOfSqs** | **MeanSqs** | **F.Model** | **R2** | **Pr(>F)** | **Significance** |
|  | 1 | 0.3926 | 0.39258 | 2.397 | 0.0908 | 0.015 | * |
| Residuals | 24 | 3.9308 | 0.16378 | - | 0.9092 | - | - |
| Total | 25 | 4.3233 | - | - | 1.00000 | - | - |
| Significance codes: 0 “***”, 0.001 “**”, 0.01 “*”, 0.05 “.”, 0.1 “”; LEP, progression score of EORTC≤6; HEP, progression score of EORTC≥7. | | | | | | | |
| **Supplementary Table 6.5\|** **PERMANOA based on unweighted UniFrac metric (LEP vs HEP)** | | | | | | | |
|  | **DF** | **SumsOfSqs** | **MeanSqs** | **F.Model** | **R2** | **Pr(>F)** | **Significance** |
|  | 1 | 0.6755 | 0.67547 | 2.0497 | 0.07868 | 0.014 | * |
| Residuals | 24 | 7.9093 | 0.32955 | - | 0.92132 | - | - |
| Total | 25 | 8.5847 | - | - | 1.00000 | - | - |
| Significance codes: 0 “***”, 0.001 “**”, 0.01 “*”, 0.05 “.”, 0.1 “”; LEP, progression score of EORTC≤6; HEP, progression score of EORTC≥7. | | | | | | | |
| **Supplementary Table 6.6\|** **PERMANOA based on Bray-Curtis metric (LEP vs HEP)** | | | | | | | |
|  | **DF** | **SumsOfSqs** | **MeanSqs** | **F.Model** | **R2** | **Pr(>F)** | **Significance** |
|  | 1 | 0.5047 | 0.50466 | 1.4647 | 0.05752 | 0.114 | - |
| Residuals | 24 | 8.2690 | 0.34454 | - | 0.94248 | - | - |
| Total | 25 | 8.7736 | - | - | 1.00000 | - | - |
| Significance codes: 0 “***”, 0.001 “**”, 0.01 “*”, 0.05 “.”, 0.1 “”; LEP, progression score of EORTC≤6; HEP, progression score of EORTC≥7. | | | | | | | |
| **Supplementary Table 6.7\|** **PERMANOA based on weighted UniFrac metric (PUNLMP vs LG vs HG)** | | | | | | | |
|  | **DF** | **SumsOfSqs** | **MeanSqs** | **F.Model** | **R2** | **Pr(>F)** | **Significance** |
|  | 2 | 0.3787 | 0.18935 | 0.76238 | 0.05164 | 0.684 | - |
| Residuals | 28 | 6.9542 | 0.24836 | - | 0.94836 | - | - |
| Total | 30 | 7.3329 | - | - | 1.00000 | - | - |
| Significance codes: 0 “***”, 0.001 “**”, 0.01 “*”, 0.05 “.”, 0.1 “”; PUNLMP, papillary urothelial neoplasm of low malignant potential; LG, low-grade papillary urothelial carcinoma; HG, high-grade papillary urothelial carcinoma. | | | | | | | |
| **Supplementary Table 6.8\|** **PERMANOA based on unweighted UniFrac metric (PUNLMP vs LG vs HG)** | | | | | | | |
|  | **DF** | **SumsOfSqs** | **MeanSqs** | **F.Model** | **R2** | **Pr(>F)** | **Significance** |
|  | 2 | 0.6171 | 0.30854 | 0.90151 | 0.0605 | 0.592 | - |
| Residuals | 28 | 9.5830 | 0.34225 | - | 0.0605 | - | - |
| Total | 30 | 10.2001 | - | - | 1.00000 | - | - |
| Significance codes: 0 “***”, 0.001 “**”, 0.01 “*”, 0.05 “.”, 0.1 “”; PUNLMP, papillary urothelial neoplasm of low malignant potential; LG, low-grade papillary urothelial carcinoma; HG, high-grade papillary urothelial carcinoma. | | | | | | | |

| **Supplementary Table 6.9\|** **PERMANOA based on Bray-Curtis metric (PUNLMP vs LG vs HG)** | | | | | | | |
| --- | --- | --- | --- | --- | --- | --- | --- |
|  | **DF** | **SumsOfSqs** | **MeanSqs** | **F.Model** | **R2** | **Pr(>F)** | **Significance** |
|  | 2 | 0.6126 | 0.30632 | 0.85067 | 0.05728 | 0.704 | - |
| Residuals | 28 | 10.0825 | 0.36009 | - | 0.94272 | - | - |
| Total | 30 | 10.6951 | - | - | 1.00000 | - | - |
| Significance codes: 0 “***”, 0.001 “**”, 0.01 “*”, 0.05 “.”, 0.1 “”; PUNLMP, papillary urothelial neoplasm of low malignant potential; LG, low-grade papillary urothelial carcinoma; HG, high-grade papillary urothelial carcinoma. | | | | | | | |
